# Functional Gradient of the Fusiform Cortex for Chinese Character Recognition

**DOI:** 10.1523/ENEURO.0495-21.2022

**Published:** 2022-05-26

**Authors:** Wanwan Guo, Shujie Geng, Miao Cao, Jianfeng Feng

**Affiliations:** 1 Institute of Science and Technology for Brain-Inspired Intelligence, Fudan University, Shanghai 200433, China; 2 Ministry of Education, Key Laboratory of Computational Neuroscience and Brain-Inspired Intelligence (Fudan University), Shanghai 200433 China

**Keywords:** Chinese character recognition, functional gradient, fusiform cortex, representational similarity analysis, univariate activation analysis

## Abstract

Visual word recognition has been proposed to have a functional and spatial organization corresponding to hierarchical language-like word forms in the left fusiform gyrus (FG) during visual word recognition in alphabetic languages. However, it is still unclear whether the similar functional gradients of word-like representation exist during Chinese character recognition. In this study, we adopted univariate activation analysis and representational similarity analysis (RSA) methods to investigate the functional organization in the FG for Chinese character recognition using task fMRI data. Native Chinese readers were visually presented with four types of character-like stimuli (i.e., real characters, pseudo-characters, false characters, and stroke combinations). After analysis, we observed a posterior-to-anterior functional gradient in the left FG corresponding to the degree of likeness of stimuli to character. Additionally, distinct subregions of the left FG harbor different orthographic codes. The middle part of the left FG was involved in abstract orthographic processing, while the anterior part of the left FG was involved in lexical orthographic processing (i.e., mapping orthography onto phonology or semantics). Notably, for the right FG, we did not find similar coding pattern for selectivity to character likeness, indicating the asymmetry of the functional hierarchical organization in favor of the left hemisphere. In conclusion, our findings revealed that the left FG presents a posterior-to-anterior gradient functional processing for Chinese character recognition, which expands our understanding of the psychological, neural, and computational theories of word reading.

## Significance Statement

The left fusiform gyrus (FG) is essential to reading, yet its functional organization during Chinese reading remains unclear. Here, we revealed a posterior-to-anterior functional gradient corresponding to the lower-to-higher character-like stimuli within the left FG during Chinese character recognition but not in its right homolog. Employing representational similarity analysis (RSA), we identified two functionally segregated subregions of the left FG: the middle part for word-form orthographic processing and the anterior part for lexical orthographic processing. For conclusion, we found the posterior, middle, and anterior regions of the left FG are responsive to distinct orthographic hierarchy thereby perform different but complementary computations. Based on this gradient pattern, the left FG interacts with other regions of language network to achieve Chinese reading.

## Introduction

Efficient visual word recognition requires a fast conversion of word form and orthography to word pronunciation and meaning (Liu, 1999; Coltheart et al., 2001; Price and Devlin, 2011). Neuroimaging and lesion studies have revealed that the left fusiform gyrus (FG) is critical for such conversion during word reading (Kuo et al., 2001; Cohen et al., 2002; Baker et al., 2007; Dehaene et al., 2010; Centanni et al., 2017). Additionally, the lateral middle region of the left FG, called the visual word form area, is thought to be spatially reproducible across different writing systems that vary greatly in the type of scripts, such as alphabetic languages (e.g., English) and logographic languages (e.g., Chinese characters; Bolger et al., 2005; Liu et al., 2008; Dehaene and Cohen, 2011). A functional hierarchical organization of word-like stimuli within the left FG during English word reading has been observed (Vinckier et al., 2007). However, whether a similar internal organization of the left FG exists in Chinese word reading is still unclear.

Recently, some studies have examined the functional organization of word-like stimuli in the ventral occipitotemporal cortex (vOT). For alphabetic languages, lines of evidence based on activation results have observed a functional and spatial hierarchical organization in the left FG during visual word recognition (Binder et al., 2006; Vinckier et al., 2007; Van der Mark et al., 2009; Kronschnabel et al., 2013; Olulade et al., 2013, 2015; Lerma-Usabiaga et al., 2018). Vinckier and colleagues found that different levels of orthographic stimuli induced equal activation in the posterior part of the left FG, whereas more word-like stimuli induced higher activation along the middle to anterior axis (Vinckier et al., 2007). Consistently, an intracranial recording study on English word recognition confirmed that the posterior part of the left FG was uniquely involved in letter selectivity, but emphasized the spatially intermingled but not strict hierarchical organization underlying prelexical and lexical responses in the middle and anterior regions of the left FG (Lochy et al., 2018). Those authors consistently identified that for the left FG, the posterior part was involved in letter processing and emphasized the functional gradient from the middle to anterior part.

Given the sharp difference between written English and Chinese in orthographic structure, two recent studies have investigated whether a similar functional gradient of brain activity for character-like stimuli exists in Chinese (Chan et al., 2009; Tian et al., 2020). Chan and colleagues found that the anterior region of the left FG was more selective for Chinese character-like stimuli with orthographic legality, whereas the posterior part was more selective for Korean characters (Chan et al., 2009). Tian and colleagues suggested that the anterior and middle regions of the left FG were more selective for radical-based stimuli, whereas the posterior region was not (Tian et al., 2020). However, the corresponding relationship between different levels of Chinese orthographic structure to subregions of the left FG has still not been clearly revealed. In addition, the right FG was also significantly activated, which was interpreted as spatial information processing during Chinese word recognition (Tan et al., 2000, 2001). However, which levels of orthography were processed and whether divergent hierarchical coding patterns existed in the right FG during Chinese word reading also remained largely unknown.

The current study examined the functional organization in the FG during Chinese character recognition by using univariate activation analysis and RSA methods. Here, we recruited a group of adults, native Chinese speakers who performed a lexical decision task for real words (RWs), pseudowords (PWs), false words (FWs), and stroke combinations (SCs) during fMRI scanning. Given that Chinese orthographic processing entails four main components: visual properties, radical orthography, word-form orthography, and lexical orthography, we hypothesize that distinct components take place in distinct subregions of the left FG, which resulting in a posterior-to-anterior gradient of Chinese orthographic processing.

## Materials and Methods

### Participants

Fifty-one college students (mean age = 23.4 years, 19–28 years old, 25 males/26 females) were recruited in the current study by online advertising. All were native Chinese speakers with normal or corrected-to-normal vision over 4.8 (Logarithmic Vision Chart Values). Forty-one were identified as right-handed, and the rest had balanced handedness according to the Edinburgh Handedness Inventory (Oldfield, 1971). None had any history of neurological disease or psychiatric disorders. Informed written consent was provided to each subject before the experiment. The current study was approved by the Ethics Committee of the School of Life Sciences, Fudan University.

### Stimuli and task fMRI procedures

**Figure 1.**
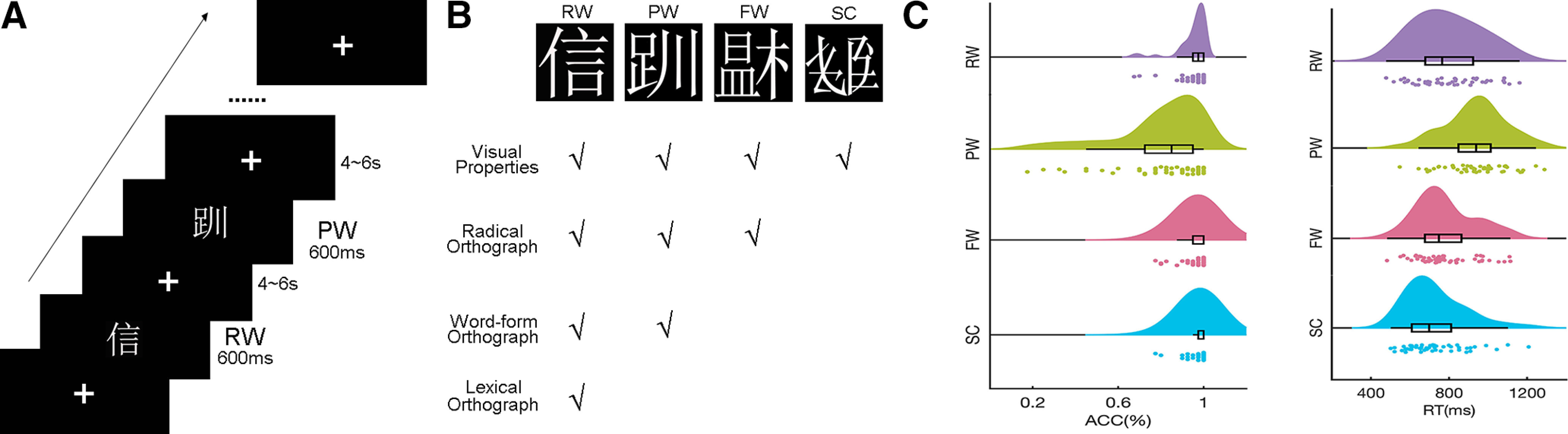
Experimental design, stimuli, and behavioral results. ***A***, Experimental design and stimuli. An event-related design and lexical decision task were adopted in the current study. ***B***, Four types of character-like stimuli were visually presented in a randomized order. In particular, these four types of stimuli represent hierarchical levels of Chinese orthography. ***C***, Behavioral results. The accuracy (ACC) and reaction time (RT) were computed for the four conditions. One-way ANOVAs were conducted to test significant differences among four types of stimuli. Error bars represent standard error. RW, real word; PW, pseudoword; FW, false word; SC, stroke combination.

The stimuli set consisted of four conditions: RWs, PWs, FWs, and SCs, with 40 trials in each condition (Fig. 1*A*). Chinese orthographic processing entails processing four putative components, that is, visual properties, radical orthography, word-form orthography, and lexical orthography, which construct a hierarchical framework of cognitive processes (Fig. 1*B*). RWs are high-frequency single-character words consisting of two radicals. PWs are formed by two radicals that are presented at their legal positions but cannot be found in the existing Chinese dictionary. Notably, in contrast with PWs in alphabetic language, PWs in Chinese are both unpronounceable and meaningless, even without phonological and semantic cues. FWs are formed by two radicals presented at illegal positions. SCs are comprised of randomly arranged strokes that appear in real characters and maintain the same envelope as real characters. The horizontal visual angle of all stimuli, which were white and presented on a black screen, was 4.37°. The percentage of pixels, picture size, and number of strokes were matched across conditions. Word frequency of RWs and single-character words used to build PWs and FWs was also matched.

In the current study, an event-related design and lexical decision task were adopted. Each stimulus was presented for 600 ms in randomized order, with a randomized interstimulus interval (ISI) ranging from 4000 to 6000 ms. A fixation cross was presented in the center of the screen during ISI to obtain baseline brain activity (Fig. 1*A*). The lexical decision task required participants to judge whether the stimulus was a real character by pressing buttons with their right index fingers. Notably, the criterion for identifying a real character was whether it has meaning or not. A practice section consisting of 16 trials (an additional four stimuli in each condition) was conducted out of the scanner before the normal experiment to ensure full understanding of task demands.

### fMRI acquisition and data preprocessing

Functional and structural magnetic resonance imaging data were collected by a 3.0-T Siemens Prisma scanner with a 32-channel head coil (Siemens Healthcare) at Zhangjiang International Brain Imaging Center (ZIC) of Fudan University, Shanghai, China. An echo planar imaging (EPI) sequence was used for functional imaging acquisition [TR = 720 ms, TE = 33 ms, flip angle = 52°, matrix size = 110 × 96, field of view (FOV) = 220 × 196 mm, slice thickness = 2 mm, number of slices = 72]. Anatomical, high-resolution, T1-weighted images were collected before tasks (TR =3000 ms, TE = 2.56 ms, flip angle = 8°, matrix size = 320 × 320, FOV = 256 × 256 mm, slice thickness = 0.8 mm, number of slices = 208).

Image preprocessing was conducted by Statistical Parametric Mapping-12 (SPM12, Wellcome Trust Centre for Neuroimaging, London, United Kingdom; http://www.fil.ion.ucl.ac.uk/spm). First, several volumes were not recorded before trigger launch to ensure T1 equilibrium. Volumes were temporally realigned to middle EPI volume and spatially realigned to correct head movement. The structural image of each subject was registered to the mean EPI image, segmented, and normalized to Montreal Neurologic Institute (MNI) space. The realigned EPI volumes were normalized to MNI space by deformation field parameters from structural image normalization. The normalized EPI volumes were smoothed with a 6 mm Gaussian kernel and high-pass filter.

### Behavioral analysis

The ACC and RT were calculated for the four conditions. The main effects of stimulus category were analyzed by one-way repeated ANOVA. Paired *t* tests with *post hoc* Bonferroni correction (*p* < 0.05) were conducted across conditions.

### Univariate activation analysis

In single subject level analysis, a general linear model (GLM) was conducted, with the convolution of stimuli onset time (SOT) and hemodynamic response function (HRF) as independent variables, the time series of fMRI signals as dependent variables and six realignment parameters as regressors. In group-level analysis, one sample *t* tests were used to analyze in each voxel to acquire activation maps for each condition [*p* < 0.05, FDR correction (*q* < 0.05), cluster size > 10].

To investigate different functional levels of FG activation during Chinese word recognition, we determined five types of brain activation maps: (1) RWs versus fixation minus PWs versus fixation for lexical effects, (2) PWs versus fixation minus FWs versus fixation for word form effects, (3) PWs versus fixation minus RWs versus fixation for abstract orthographic processing, (4) FWs versus fixation minus RWs versus fixation for low-level orthographic processing, and (5) SCs versus fixation minus RWs versus fixation for basic visual processing. Specifically, PWs have the same orthographic regularity as RWs but fail to access lexical phonology and meaning. FWs have regular radicals or logo-graphemes but no legal Chinese orthography while SCs were spatially interleaved. Together, the functional level is incremental from the first to the fifth contrasts. Besides, less processing stages but more activation were expected for the later three contrasts because of the prediction errors because of the last failed stage, i.e., the stronger activation for more attempts to map global orthography onto word phonology and meaning or to integrate local radicals into a whole character (Price and Devlin, 2011).

### RSA

RSA is powerful for integrating different level/scale/modality (e.g., neural, behavioral, physical, theoretical) activities to identify cognitive manipulation (Fischer-Baum et al., 2017; Wang et al., 2018; Deniz et al., 2019). The current study aimed to investigate the precise functional roles of the FG during Chinese word recognition. This goal was achieved by relating the theoretical representational dissimilarity matrix (RDM) of different levels of Chinese orthography and neural RDM in the FG. Quantifying dissimilarities between abstract and lexical orthography is the key question. We achieved this result by calculating the logo-grapheme representations of RWs, PWs, and FWs.

#### Theoretical RDMs

The logo-grapheme is the basic representational unit of Chinese characters (Han et al., 2007). The logo-grapheme RDM was constructed by calculating one minus the ratio of shared basic units between any two stimuli within RWs, PWs, and FWs. Note that SCs consist of random strokes, but not all strokes are logo-graphemes. Thus, logo-grapheme RDMs can only be constructed for RWs, PWs, and FWs. Logo-grapheme representations indicate internal manipulations treating the logo-grapheme as the minimum unit. During character recognition, internal cognitive processes contain lexical orthography (i.e., orthographic legality and mapping word form onto phonology and semantics), word-form orthography (i.e., radical position and orthographic legality), radical orthography (i.e., stroke position), and general visual information composed of light and dark patches. During PW recognition, the logo-grapheme representations indicate processing orthographic legality and general visual properties. For FW recognition, the logo-grapheme representations indicate radical and general visual processing.

Semantic representations were calculated for RWs, as PWs and FWs were meaningless. Semantic dissimilarity was calculated as one minus the cosine similarity between word vectors of any pair of RW stimuli. Skip-gram algorithms (window size = 5, subsampling rate = 10 − 4, negative sample number = 5, learning rate = 0.025, dimension number = 300) were used to calculate word vectors based on the open-source Wikipedia Chinese Corpus.

#### Neural RDMs and searchlight RSA

A GLM was performed at the first level for each of 120 trials, with 6 head motion parameters regressed. In each condition (RWs, PWs, and FWs) and for each subject, voxel-wise neuronal similarities between any pair of 40 trials were calculated as significant correlations between β-values extracted from a self-centered sphere with a 6-mm radius. A one minus correlation between any two stimuli was set as the dissimilarity. The centered voxel of the sphere completed transversally within cortical regions of interest (ROIs), such as a searchlight, and voxel-wise neural RDMs were obtained for each subject in each condition. The ROIs in the current study were defined as the bilateral fusiform areas (55#, 56#) in the Automated Anatomical Labeling 3 (AAL3) template. Bilateral inferior occipital cortices (53#, 54#) in AAL3 were also included. Spearman’s correlations were calculated between neural RDMs and logo-grapheme/semantic RDMs at the voxel level. Spearman’s ρ transformed Z values were logo-grapheme/semantic representation values and used to perform a one-tailed, one-sample *t* test across subjects at the voxel level. Significant voxels (*p* < 0.05, uncorrected, cluster size > 10) in the *t* test were identified as involved in logo-grapheme/semantic representation. The analysis scripts and the summary data are available at GitHub (http://github.com/miaocao88/Functional-Gradient-in-vOT).

### Validation analysis

To examine whether behavioral performance (ACC) affects brain activity during lexical decision task, validation analysis was conducted by excluding trials in which participants inaccurately judged the lexicality. Particularly, for PWs condition, 6 participants whose ACC is <50% were excluded to ensure statistical effect of RSA results.

## Results

### Behavioral results

The ACC and RT of button pressing for the lexical decision task were analyzed. The main effects of ACC and RT among RWs, PWs, FWs, and SCs calculated by one-way repeated ANOVA were both significant, as shown in Figure 1*B* (Allen et al., 2019). Significant main effects measured by one-way repeated ANOVA were observed for both ACC (*F*_(3,150)_ = 27.12, *p* < 0.001) and RT (*F*_(3,150)_ = 16.68, *p* < 0.001). The ACC of PWs (0.80 ± 0.21) was significantly lower than that of RWs (0.95 ± 0.07, *t*_(50)_ = −5.29, *p* < 0.001, Bonferroni corrected), FWs (0.96 ± 0.06, *t*_(50)_ = −6.12, *p* < 0.001, Bonferroni corrected), and SCs (0.98 ± 0.05, *t*_(50)_ = −6.23, *p* < 0.001, Bonferroni corrected), whereas the RT of PWs (938.81 ± 15.60 ms) was significantly higher than that of RWs (793.78 ± 170.21 ms, *t*_(50)_ = 9.04, *p* < 0.001, Bonferroni corrected), FWs (780.41 ± 149.84 ms, *t*_(50)_ = 10.28, *p* < 0.001, Bonferroni corrected), and SCs (728.68 ± 152.54 ms, *t*_(50)_ = 12.84, *p* < 0.001, Bonferroni corrected). The ACC of SCs was greater than that of RWs (*t*_(50)_ = 2.89, *p* < 0.05, Bonferroni corrected). The RT of SCs was shorter than that of FWs (*t*_(50)_ = −4.85, *p* < 0.001, Bonferroni corrected) and RWs (*t*_(50)_ = −5.30, *p* < 0.001, Bonferroni corrected). Together, subjects showed poorest performance in PW recognition compared with the other three conditions but better performance for SCs in the lexical decision task.

### Functional activation results

**Figure 2.**
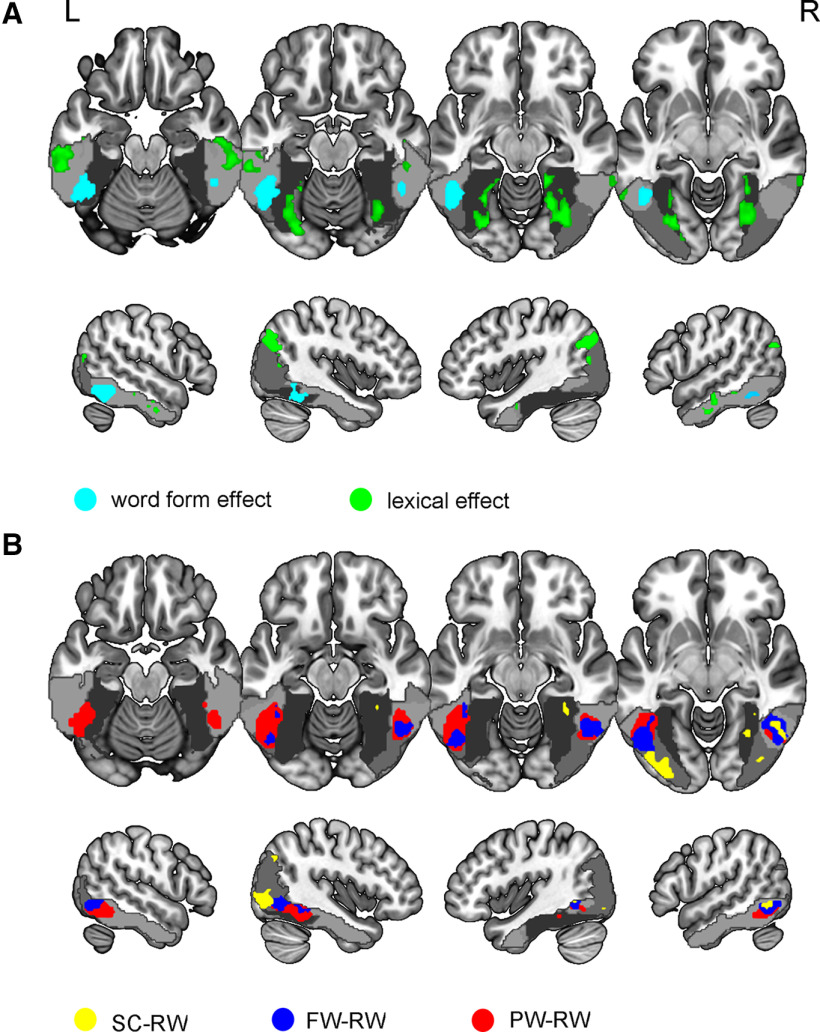
Activations induced by five types of contrasts in the left and right FG. ***A***, Activations induced by lexical effects and word form effects in the FG. RWs versus fixation minus PWs versus fixation indicates lexical effect. PWs versus fixation minus FWs versus fixation indicates word form effect. ***B***, Activation induced by the three types of stimuli minus RWs in the FG. The activation response induced by PWs versus fixation minus RWs versus fixation was involved in word-form orthographic processing. Activation response induced by FWs versus fixation minus RWs versus fixation represented radical orthographic processing. The activation response induced by SCs versus fixation minus RWs versus fixation indicated basic visual processing. Light gray indicates the inferior temporal gyrus. Middle gray indicates the middle occipital gyrus. Dark gray denotes the FG. See Extended Data Figure 2-1. RW, real word; PW, pseudoword; FW, false word; SC, stroke combination; *p* < 0.05, FDR correction (*q* < 0.05), cluster size > 10.

In the current study, the word form effect was defined as activation of PWs versus fixation minus FWs versus fixation, whereas the lexical effect was defined as RWs versus fixation minus PWs versus fixation. As shown in Figure 2*A*, the word form effect activated the bilateral ventral occipitotemporal cortices and left middle occipital gyrus [*p* < 0.05, FDR correction (*q* < 0.05), cluster size > 10]. Left word form effect areas were located in a large cluster (cluster size = 472) spanning the middle part of the left lateral occipitotemporal sulcus, including the left inferior temporal gyrus, middle and anterior parts of the left FG and left inferior occipital gyrus [*p* < 0.05, FDR correction (*q* < 0.05), cluster size > 10]. Right word form effect areas involved the contralateral homotopic cortices, including the right inferior temporal gyrus and middle FG. The lexical effect activated extensive brain regions, including the bilateral middle occipital gyrus, bilateral occipitotemporal cortices (consisting of the inferior temporal gyrus and middle FG), right FG, and anterior part of the left inferior temporal gyrus [*p* < 0.05, FDR correction (*q* < 0.05), cluster size > 10]. Massively activated brain regions might be derived from top-down modulation of lexical responses. Note that more anterior activations of lexical effects were found in the anterior part of the left inferior temporal gyrus than in the anterior part of the left FG. For more details, please see Table 1.

**Table 1:** Main activation clusters and peaks of the lexical effect and word form effect as identified by contrasting RWs versus fixation minus PWs versus fixation and PWs versus fixation minus FWs versus fixation

Region	Cluster size	Peak *t* value	Peak *p* value	Peak coordinates		
				*x*	*y*	*z*
Lexical effect: RWs vs fixation minus PWs vs fixation	
Right middle occipital gyrus, right middle temporal gyrus,	310	6.80	<0.001	44	−72	32
Left middle occipital gyrus, left middle temporal gyrus	381	5.91	<0.001	−42	−78	38
Right inferior temporal gyrus, right FG, right middle temporal gyrus	409	5.32	<0.001	60	−22	−26
Left middle occipital gyrus	141	5.07	<0.001	−18	−90	18
Left inferior temporal gyrus, left middle temporal gyrus, left FG	421	5.01	<0.001	−58	−24	−24
Left FG, left inferior occipital gyrus	358	4.74	<0.001	−26	−78	−12
Left middle temporal gyrus, left inferior temporal gyrus	28	4.43	<0.001	−64	−58	−4
Right FG	458	4.36	<0.001	18	−44	−12
Left middle temporal gyrus	11	3.73	<0.001	−52	−72	18
Right middle occipital gyrus	35	3.69	<0.001	40	−72	14
Left inferior temporal gyrus	22	3.63	<0.001	−48	6	−40
Right middle occipital gyrus	47	3.29	0.001	26	−86	14
Right inferior temporal gyrus	33	3.26	0.001	68	−46	−8
Left inferior temporal gyrus	12	2.84	0.003	−42	−68	10
Word form effect: PWs vs fixation minus FWs vs fixation
Left inferior temporal gyrus, left FG, left inferior occipital gyrus	472	5.74	<0.001	−50	−50	−14
Right inferior temporal gyrus, right FG	43	4.14	<0.001	50	−46	−18
Left middle occipital gyrus	12	3.80	<0.001	−28	−68	40

See Extended Data Table 1-1.

10.1523/ENEURO.0495-21.2022.f2-1 Extended Data Figure 2-1 Validation for functional activation results, supporting Figure 2. Download Figure 2-1, TIF file.

Based on the prediction error hypothesis, PWs versus fixation minus RWs versus fixation, FWs versus fixation minus RWs versus fixation and SCs versus fixation minus RWs versus fixation corresponded to abstract orthographic processing, radical processing, and visual properties extraction, respectively, which belong to higher-to-lower levels of orthographic structure. As shown in Figure 2*B*, PWs versus fixation minus RWs versus fixation activated the bilateral ventral occipitotemporal cortices and bilateral middle occipital gyrus [*p* < 0.05, FDR correction (*q* < 0.05), cluster size > 10]. Brain regions for FWs versus fixation minus RWs versus fixation were found in the bilateral inferior temporal gyrus and left middle occipital gyrus [*p* < 0.05, FDR correction (*q* < 0.05), cluster size > 10]. SCs versus fixation minus RWs versus fixation only activated the left middle and inferior occipital gyrus [*p* < 0.05, FDR correction (*q* < 0.05), cluster size > 10]. Gradually changed and intermingled activations along the *y*-axis in the posterior part of the left inferior temporal gyrus are shown in the lowest panel of Figure 2*B* and confirmed functional gradients of the left FG. For more details, please see Table 2.

**Table 2:** Mean activations clusters and peaks as identified by contrasting PWs versus fixation minus RWs versus fixation, FWs versus fixation minus RWs versus fixation, and SCs versus fixation minus RWs versus fixation

Region	Cluster size	Peak *t* value	Peak*p* value	Peak coordinates		
				*x*	*y*	*z*
Word-form orthographic processing: PWs vs fixation minus RWs vs fixation
Left inferior temporal gyrus, left inferior occipital gyrus, left FG	828	8.82	<0.001	−48	−64	−10
Right inferior temporal gyrus, right FG, right inferior occipital gyrus	408	6.51	<0.001	48	−54	−12
Left middle occipital gyrus	91	5.00	<0.001	−24	−60	42
Right middle occipital gyrus	20	4.28	<0.001	30	−64	36
Radical orthographic processing: FWs vs fixation minus RWs vs fixation
Left middle occipital gyrus, left inferior temporal gyrus,	394	5.73	<0.001	−46	−68	−8
Right inferior temporal gyrus	289	5.38	<0.001	46	−56	−6
Basic visual processing: SCs vs fixation minus RWs vs fixation
Left middle occipital gyrus, left inferior occipital gyrus	127	5.31	<0.001	−42	−82	−4

See Extended Data Table 2-1.

### RSA results

**Figure 3.**
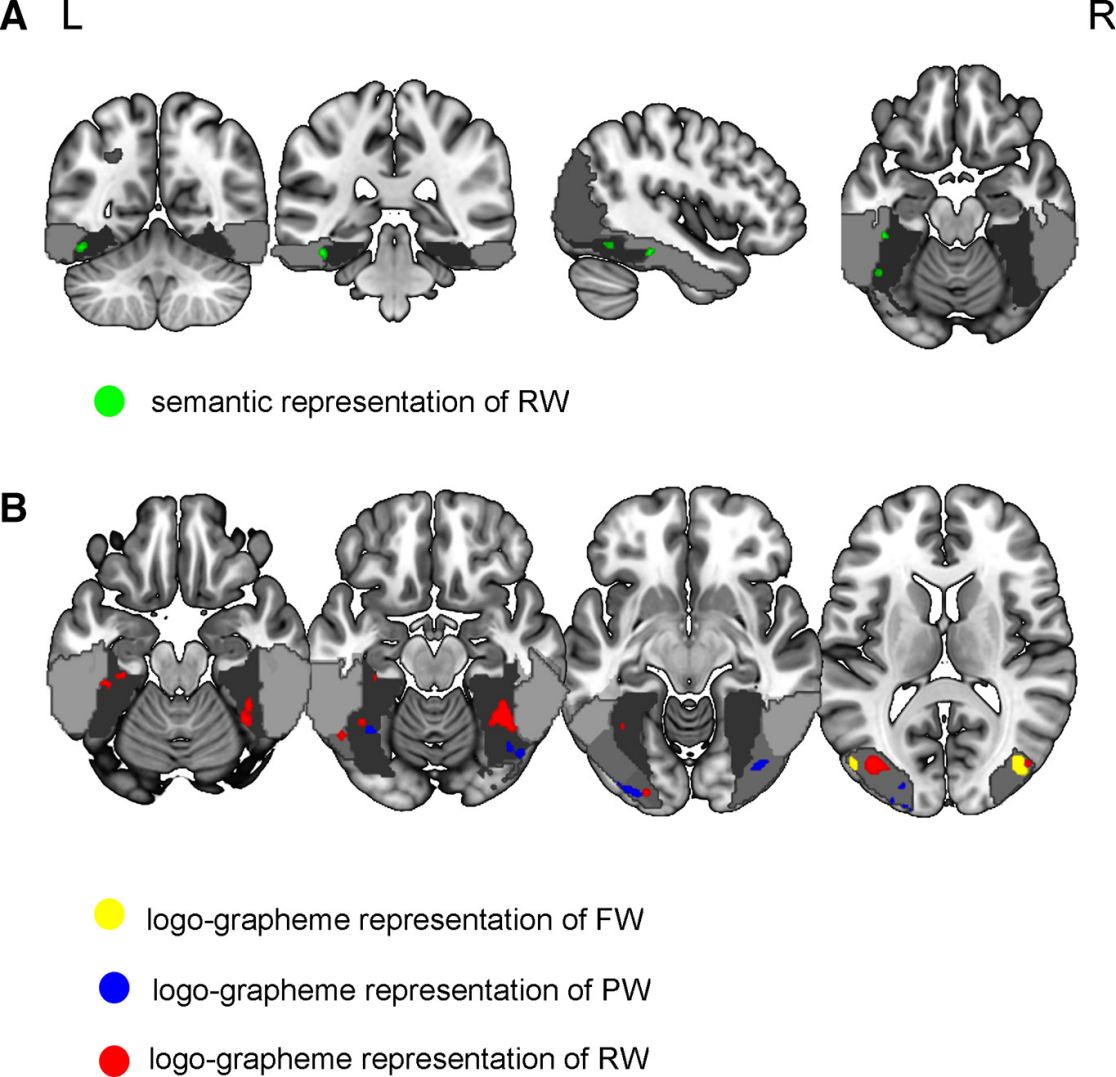
Neural representations of RWs, PWs, and FWs in the vOT. ***A***, Semantic representations of RWs in the vOT. ***B***, Logo-grapheme representations of RWs, PWs, and FWs in the vOT. Light gray indicates the inferior temporal gyrus. Middle gray indicates the middle occipital gyrus. Dark gray denotes the FG. See Extended Data Figure 3-1. RW, real word; PW, pseudoword; FW, false word.

Semantic representations were only explored for RWs recognition, resulting in two clusters, the left middle and anterior FG, both of which were close to the lateral occipitotemporal sulcus (Fig. 3*A*).

10.1523/ENEURO.0495-21.2022.tab1-1 Extended Data Table 1-1 Main activation clusters and peaks of the lexical effect and word form effect as identified by contrasting RWs versus fixation minus PWs versus fixation and PWs versus fixation minus FWs versus fixation by removing trials with error response. This table is supporting the Table 1. Download Table 1-1, DOCX file.

10.1523/ENEURO.0495-21.2022.tab2-1 Extended Data Table 2-1 Mean activations clusters and peaks as identified by contrasting PWs versus fixation minus RWs versus fixation, FWs versus fixation minus RWs versus fixation, and SCs versus fixation minus RWs versus fixation by removing trials with error response. This table is supporting the Table 2. Download Table 2-1, DOCX file.

10.1523/ENEURO.0495-21.2022.f3-1 Extended Data Figure 3-1 Validation for RSA results, supporting Figure 3. Download Figure 3-1, TIF file.

Logo-grapheme representation is involved in cognitive processing of orthography, radicals, and composite visual features, which were explored for FWs, PWs and RWs recognition. FWs recognition did not include legal orthography, and the logo-grapheme representations of FWs were found in the bilateral middle occipital gyrus (Fig. 3*B*). In addition to the bilateral middle and inferior occipital gyrus, the logo-grapheme representations of PWs were also found in the left middle FG (Fig. 3*B*), which may serve as abstract orthography because of the lack of semantics in PWs. The logo-grapheme representations of RWs were observed in the bilateral middle occipital gyrus, left inferior occipital gyrus, bilateral middle FG and left anterior FG (Fig. 3*B*). Particularly, the left middle and anterior FG were both involved in orthographic representations of RWs, but only the left middle FG participated in those of PWs, suggesting that the middle and anterior parts of the left FG have different functional roles. The left middle FG induced abstract orthography and the left anterior FG were related to lexical orthography. For more details, please see Table 3.

**Table 3:** Clusters and peaks for logo-grapheme and semantic representations of RWs, PWs, and FWs in the vOT

Index	Region	Cluster size	Peak coordinates		
			*x*	*y*	*z*
Semantic representations of RWs
1	Left FG	10	−42	−34	−20
2	Left FG	18	−44	−56	−16
Logo-grapheme representations of RWs	
1	Left middle occipital Gyrus	141	−14	−102	4
2	Right middle occipital gyrus	14	32	−68	24
3	Left FG	44	−30	−30	−22
4	Right FG	108	36	−52	−16
5	Left middle occipital gyrus	69	−38	−76	12
6	Right middle occipital gyrus	61	34	−80	28
7	Left inferior occipital gyrus	10	−50	−62	−16
8	Right middle occipital gyrus	12	46	−78	10
9	Left middle occipital gyrus	13	−24	−84	22
10	Left FG	13	−38	−54	−16
Logo-grapheme representations of PWs
1	Left middle occipital Gyrus, left inferior Occipital gyrus	96	−36	−92	−4
2	Right inferior occipital gyrus, right middle occipital gyrus	176	40	−90	−4
3	Left middle occipital gyrus	120	−32	−92	22
4	Left middle occipital gyrus	36	−36	−74	40
5	Left FG	12	−34	−60	−16
6	Right inferior occipital gyrus	14	30	−92	−6
7	Right inferior occipital gyrus	15	46	−70	−18
Logo-grapheme representations of FWs
1	Right middle occipital gyrus	131	42	−80	8
2	Left middle occipital gyrus	26	−48	−80	14
3	Left middle occipital gyrus	10	−36	−82	8
4	Right middle occipital gyrus	14	−48	−80	14

See Extended Data Table 3-1.

**Figure 4.**
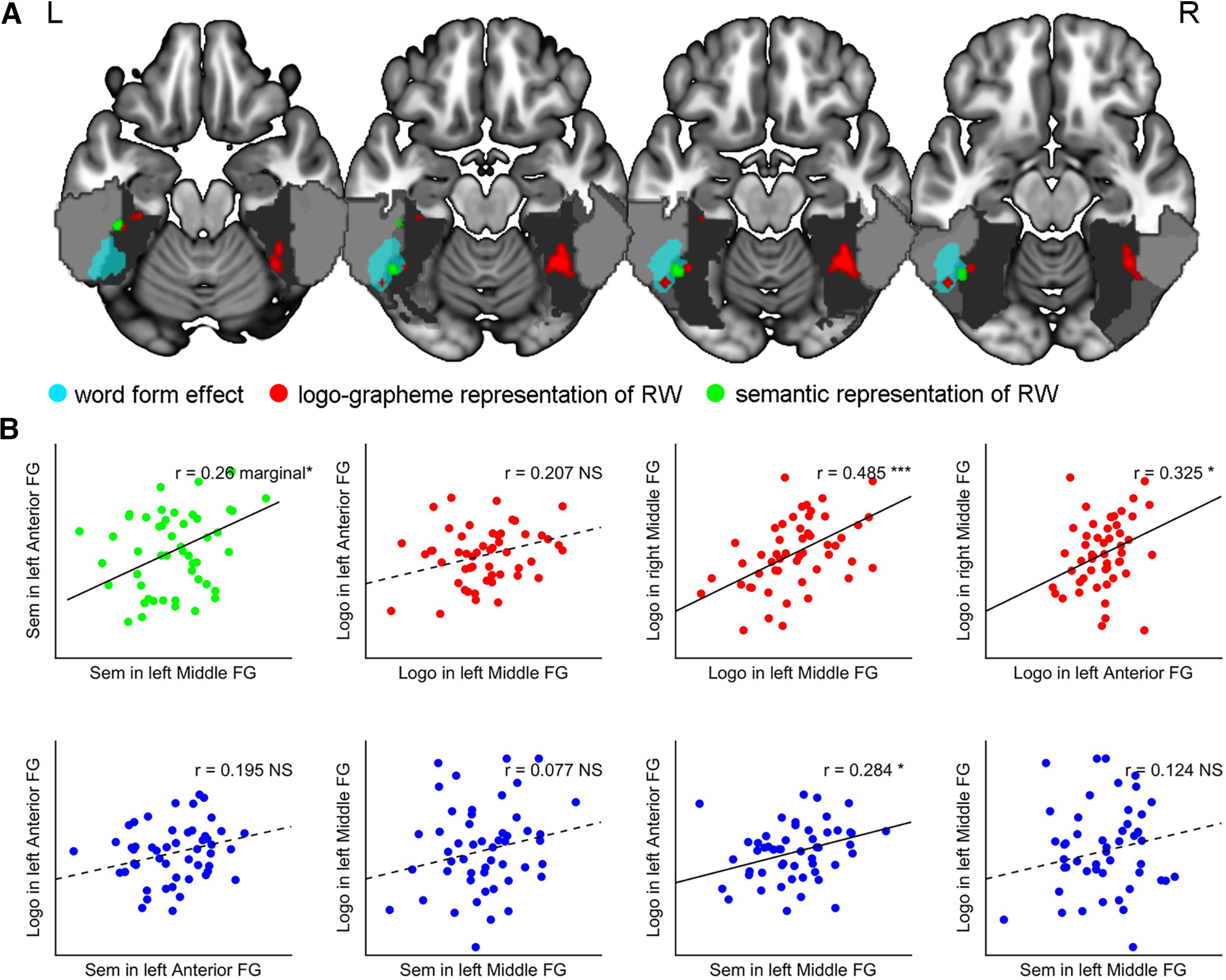
Logo-grapheme and semantic representations of RWs in the left middle and anterior FG and correlations between the left middle and anterior FG. ***A***, Logo-grapheme and semantic representations of RWs and word form effect areas in the vOT. ***B***, Correlations of logo-graphemes and semantic representations of RWs between the left middle and anterior fusiform regions. Solid line indicates that the correlation is significant, and dash line indicates that the correlation is not significant. Light gray indicates the inferior temporal gyrus. Middle gray indicates the middle occipital gyrus. Dark gray denotes the FG. See Extended Data Figure 4-1. RW, real word; NS, not significant; *p < 0.05, ***p < 0.001; marginal*p = 0.0661.

Notably, during RWs recognition, the logo-grapheme and semantic representations were observed in both the left middle and anterior FG and along with the lateral occipitotemporal sulcus. Clusters underlying logo-grapheme and semantic representations spatially neighbored each other in the left middle and anterior FG, respectively. To explore the relationships among the logo-grapheme and semantic representations between the left middle and anterior FG, Spearman’s correlation analysis was conducted across subjects (Fig. 4*B*). A marginally significant correlation was found between the semantic representations in the left middle fusiform and left anterior fusiform regions (*r* = 0.26, *p* = 0.067). Logo-grapheme representations in the right middle FG were significantly correlated with the logo-grapheme representations in the left middle (*r* = 0.485, *p* < 0.001) and anterior FG (*r* = 0.325, *p* = 0.020). Logo-grapheme representations of the left anterior FG were significantly correlated with the sematic representations of the left middle FG (*r* = 0.284, *p* = 0.044). No significant correlation between the logo-grapheme representations in the left middle FG and left anterior FG were detected. Notably, as shown in Figure 4*A*, clusters underlying logo-grapheme and semantic representations in the left anterior FG and clusters in the left middle FG were neighbored or next to the anterior and posterior part of word form effect areas which were discovered during the activation analysis. But for the lexical effect areas, no overlapping was found within the areas of logo-grapheme and semantic representations of RWs.

10.1523/ENEURO.0495-21.2022.tab3-1 Extended Data Table 3-1 Clusters and Peaks for logo-grapheme and semantic representations of RWs, PWs, and FWs in the vOT by removing trials with error response. This table is supporting the Table 3. Download Table 3-1, DOCX file.

10.1523/ENEURO.0495-21.2022.f4-1 Extended Data Figure 4-1 Validation for RSA-behavior correlation results, supporting Figure 4. Download Figure 4-1, TIF file.

### Validation results

As shown in Extended Data Figures 2-1, 3-1, 4-1 and Extended Data Tables 1-1, 2-1, 3-1, both the activation and RSA results based on data after excluding are consistent with the results based on all data, indicating that behavioral performance might have little effect on brain response of participants.

## Discussion

In the current study, we aimed to investigate the functional gradient within the FG corresponding to different levels of orthographic structure in a visual lexical decision task to recognize four types of character-like stimuli. Different from the univariate analysis which identifies brain response to experimental stimulus through linearly fitting the behavior response with the hemodynamic activities of brain voxels, RSA characterizes the correspondence between brain activity patterns and theoretical/behavioral measurement (e.g., neural, behavioral). Therefore, although both these two methods can characterize brain activities, RSA can detect more fine-grained pattern information than the univariate analysis. Our activation-based and RSA results revealed that there was a posterior-to-anterior gradient for orthographic processing of character-like stimuli within the left FG. Besides, three functionally segregated regions within the left FG, a posterior, a middle, and an anterior region, were detected while no similar pattern was observed in the right FG. These findings revealed the neural basis for preprocessing of the hierarchical framework of Chinese orthography, i.e., general visual properties, radical orthography, orthography, and lexical orthography.

### Functional gradients of character selectivity within the left FG

Although previous research revealed the involvement of the left FG for visual word recognition (Cohen et al., 2002; Bruno et al., 2008; Glezer et al., 2009, 2015; Baeck et al., 2015; Lochy et al., 2018), the levels of orthographic structure for the left FG involvement have not been clearly elucidated (Kuo et al., 2004; Liu et al., 2008; Price and Devlin, 2011). Our results showed that the left occipitotemporal cortex preferentially responds to orthographically legal characters (i.e., RWs and PWs), which were consistent to previous findings (Price et al., 1996; Cohen et al., 2002; Ben-Shachar et al., 2007; Vinckier et al., 2007; Chan et al., 2009; Tian et al., 2020; Liu et al., 2021). Additionally, based on the minimum difference in orthographic legality between PWs and FWs, we found a word-form effect in the left middle FG, indicating the selectivity to orthographic legality, i.e., radical position for character identification (Wu et al., 2012). Furthermore, a lexical effect in the anterior part of the left FG were observed based on the minimum difference in lexical orthography between RWs and PWs, which indicated that the anterior part of the left FG may integrate phonological or semantic information from higher level cortical areas such as the left angular gyrus, left supramarginal gyrus, and left inferior frontal gyrus, possibly through the arcuate fasciculus (Price et al., 2003; Liu et al., 2021).

Besides, we observed that PWs elicited more activations in the left middle FG, which were consistent with previous findings (Fiez et al., 1999; Xu et al., 2001). Meanwhile, FWs induced more activation in the posterior part of the left FG, while SCs elicited more activation in the left middle occipital gyrus. These findings support the prediction error hypothesis, which means that when a stimulus is recognized as potentially meaningful but is not predicted by its visual word form efficiently, it may elicit increased brain activity (Price and Devlin, 2011; J Zhao et al., 2019; Gagl et al., 2020). In line with previous findings in alphabetic languages, the varied activation patterns also revealed the corresponding relationship between functional gradient of the left FG and similarity to RWs, indicating the attuning to orthographic regularities of the reader’s language in the course of learning to read (Vinckier et al., 2007).

### Functional segregation of subregions in the left FG

To further examine the functional roles of the subregions of the left FG, we investigated the logo-grapheme representations of RWs, PWs, and FWs by using RSA methods. We observed that the logo-grapheme representations of RWs were detected in the middle and anterior parts of the left FG, whereas the logo-grapheme representations of PWs were only in the left middle FG, which might because of the difference between cognitive processing of RWs and PWs. These findings indicated that the left middle FG was processing word-form orthography, whereas the anterior part of the left FG was involved in lexical orthographic processing. Notably, in line with prior findings, we found that semantic representations in the left anterior FG and logo-grapheme representations in the left middle FG were well aligned with the anterior and posterior part of word form selective areas, respectively, indicating the functional subdivisions of left FG (Lerma-Usabiaga et al., 2018; White et al., 2019). Besides, the logo-grapheme representations of FWs were detected in the posterior region of the left FG. Therefore, despite highly discriminated linguistic features between Chinese and English (Mo et al., 2015), a similar functional gradients of the left FG exist for both Chinese and alphabetic languages processing, which indicating a radical-based stimulus scale in Chinese characters, like the letter-based stimulus scale in alphabetic languages (Vinckier et al., 2007; Lochy et al., 2018).

To identify the gradient of abstract orthography to lexical orthography from the middle part to the anterior part of the left FG, we also calculated the correlations between the brain representations of RWs. No significant correlation was found for logo-grapheme representations between the middle and anterior parts of the FG, which may imply that there are two different types of orthographic processing represented in the middle and anterior parts of the left FG. Meanwhile, a significant correlation between the logo-grapheme representations of the anterior part of the left FG and semantic representations of the middle part of the left FG was observed, which implied that the anterior region of the left FG might integrate semantic information from the left middle FG through top-down modulation to process orthography. Previous studies have revealed the existence of top-down modulation from high-level regions such as the left inferior frontal gyrus and left middle and superior temporal gyrus to the left middle FG (LB Zhao et al., 2017; Lerma-Usabiaga et al., 2018; Wang et al., 2018; Liu et al., 2021).

In general, both results of univariate activation analysis and RSA analysis confirmed functional gradients in the left FG but not the right FG during Chinese word recognition (Figs. 2, 3). Furthermore, RSA analysis provided more fine-grained results by voxel-wise decomposing cognitive components (logo-grapheme and semantics) of each task condition. Logo-grapheme representations and semantic representations of RWs in left middle FG were included in word-form effect area (Fig. 4*A*), which implied more than one cognitive process within a single functional gradient collectively supported its linguistic function. Potential associations between semantic representations in the left middle and anterior FG (Fig. 4*B*) showed possible interactions of cognitive components between different functional gradient. Future study should focus on how functional gradients in the left FG is organized by investigating complex interactions of cognitive components within and between gradients.

### Functional organization of character selectivity in the right FG

Because of the square shape of Chinese characters, substantial evidence has shown that the right FG is specifically involved in Chinese character recognition to process spatial information such as the locations of different strokes and radicals composing the character (Tan et al., 2000, 2001, 2005; Bolger et al., 2005; Guo and Burgund, 2010). We also found that not only real characters but pseudo-characters and false characters all elicited activation of the right FG. However, we did not find a hierarchical functional organization of Chinese orthography in the right FG, which was in line with previous findings (Vinckier et al., 2007; Chan et al., 2009; Kronschnabel et al., 2013; Olulade et al., 2013; LB Zhao et al., 2017; Tian et al., 2020). Given that the right FG was proposed to process radical configuration or visual-spatial information (Peyrin et al., 2006; Deng et al., 2011; Woodhead et al., 2011), character-like stimuli comprising strokes or radicals packed into a square shape may elicit similar activation patterns in the right FG. Additionally, it was indicated that the left FG stores information in terms of parts and their relationships to visual objects, whereas the right FG stores holistic information about visual objects (Dien, 2009).

Furthermore, neither a functional gradient of the logo-grapheme representations for character-like stimuli nor semantic representation were found for the right FG, which may indicate that the right FG was only involved in visual spatial processing rather than lexical processing during Chinese character recognition. Notably, we found significant correlations between the logo-grapheme representation of RWs in the right middle FG and that of RWs in the left middle and anterior FG. Several lesion studies have proposed that the splenium of the corpus callosum links the left FG to its right homolog, thereby integrating visual information projected to bilateral visual areas (Binder and Mohr, 1992; Molko et al., 2002; Shan et al., 2010). Our results suggested that the orthographic representations of Chinese characters may integrate visual spatial information from the right middle FG and orthographic information from the left FG.

Two limitations of this study should be addressed. First, although we speculated that the anterior region of left FG may receive top-down modulation from higher-level brain regions such as the left inferior frontal gyrus and left superior and middle temporal gyrus, the present study could not provide direct evidence for this implication because of the limitations of the temporal resolution of fMRI. Future studies employed other imaging methods should be conducted to test this assumption. In addition, recent intra-cranial recordings study has suggested that functional gradient within the left FG may represent varying degrees of top-down influence from the left middle FG to primary visual cortex (Woolnough et al., 2021), which further emphasizes the importance of multiple modality studies in the future. Second, our data cannot determine whether subregions within the fusiform cortex are involved in bottom-up only or interactive bottom-up and top-down processes, as stated by two of the main theoretical proposals regarding the functional role of this region. Future studies exploring the interactions among orthography and higher-level linguistic processes would be helpful for this question (i.e., phonology and semantics).

In conclusion, we observed a posterior-to-anterior functional gradient of character-like stimuli with increasing sensitivity from SCs to real characters within the left fusiform cortex but not in its right homolog. Based on RSA results, we identified that the left middle FG was involved in word form orthographic processing, while the anterior part of left FG was involved in lexical orthographic processing. These findings indicated that the left fusiform cortex presents a posterior-to-anterior gradient corresponding to the lower-to-higher likeness of character type during Chinese character recognition.

## Synthesis

Reviewing Editor: Macià Buades-Rotger, University of Luebeck Decisions are customarily a result of the Reviewing Editor and the peer reviewers coming together and discussing their recommendations until a consensus is reached. When revisions are invited, a fact-based synthesis statement explaining their decision and outlining what is needed to prepare a revision will be listed below. The following reviewer(s) agreed to reveal their identity: Oscar Woolnough, Li Hai Tan. Note: If this manuscript was transferred from JNeurosci and a decision was made to accept the manuscript without peer review, a brief statement to this effect will instead be what is listed below.

Dear authors,

Thank you for your work on fusiform gradients in character recognition. The study is well-motivated, well-designed, well-analyzed and well-powered, and builds on prior, established fMRI findings that show an apparent visual complexity gradient with increasing word-likeness, in Latin scripts, through ventral visual cortex (e.g. Vinckier et al. 2007). The current study expands on this work to robustly show comparable results for Chinese characters, including demonstrating this gradient is strongly left-lateralized, as in alphabetic scripts. The main concerns to be addressed are detailed in the following:

- Please make the data and scripts publicly available and provide a data availability statement in the paper.

- It would be helpful to recall the acronyms of the different conditions at the beginning of the results section.

- Results: please provide exact p-values rather than approximations.

- The authors should explicitly address whether and how the univariate and RSA analyses differ or agree with each other in the Discussion.

- The presented left hemisphere word-form cluster and the more anterior lexical effect cluster (Figure 2A) appear to align well with expected locations and functional characteristics of VWFA-1 (perceptual) and VWFA-2 (lexical) (Lerma-Usabiaga et al. 2018 PNAS; White et al. 2019 PNAS). Further discussion in the context of these results would position the current work more strongly in the recent literature.

- While fMRI complexity gradients have been used to advocate a feedforward build-up of complexity through the visual system, recent intracranial work suggests this fMRI derived gradient may actually represent varying degrees of top-down influence from middle FG to earlier visual cortex (Woolnough et al. 2021 Nature Human Behaviour). These results could be incorporated into the limitations where some of these issues are currently discussed.

- The authors detail how participants performed a lexical decision task with each presented stimulus. Were any trials excluded due to behavioral performance? The results may be further strengthened by excluding trials in which participants inaccurately judged the lexicality.

- In Figure 1C the authors display the population performance statistics for the behavioral task using bar graphs with error bars. The authors should consider using a plot type that better represents the data variability such as a Raincloud plot (Allen et al. 2019 Wellcome Open Research). There also appears to be an erroneous second 0% y-axis marker in the accuracy plot.

- In Figure 2A it is unclear why the authors have separated the activation clusters for the left and right hemispheres onto different brain volumes for the axial slices. These should all be presented within the same brains, as in Figures 3 and 4.

- In Figure 4B one of the significances is annotated as “marginal”. This should either be marked exclusively with a * to denote p<0.05 or have the exact p-value presented.

Minor

- Abstract: “For conclusion” is incorrect. “In conclusion” would be more appropriate. “Expanding” -> Expands

- Significance statement: “Employing representational similarity analysis method” -> “method” can be deleted

- Line 49: “the lateral middle region of the left FG, called the visual word form area, was thought to be spatially reproducible across different writing systems”. WAS thought? Is that not the case anymore?

- In Line 64 there is a typo “prelexcial"

- In line 89, could the authors include the upper and lower limits of the age range included?

- In Line 90, the authors state that participants had “vision/corrected vision over 4.8”. Could they clarify which measurement system was used as it is unclear which visual acuity measure this value relates to?
