# Supplementary figures and images for: Functional Gradient of the Fusiform Cortex for Chinese Character Recognition
Source: eNeuro. 2022 May 26;9(3):ENEURO.0495-21.2022. doi: 10.1523/ENEURO.0495-21.2022 (PMC9172282; doi:10.1523/ENEURO.0495-21.2022)

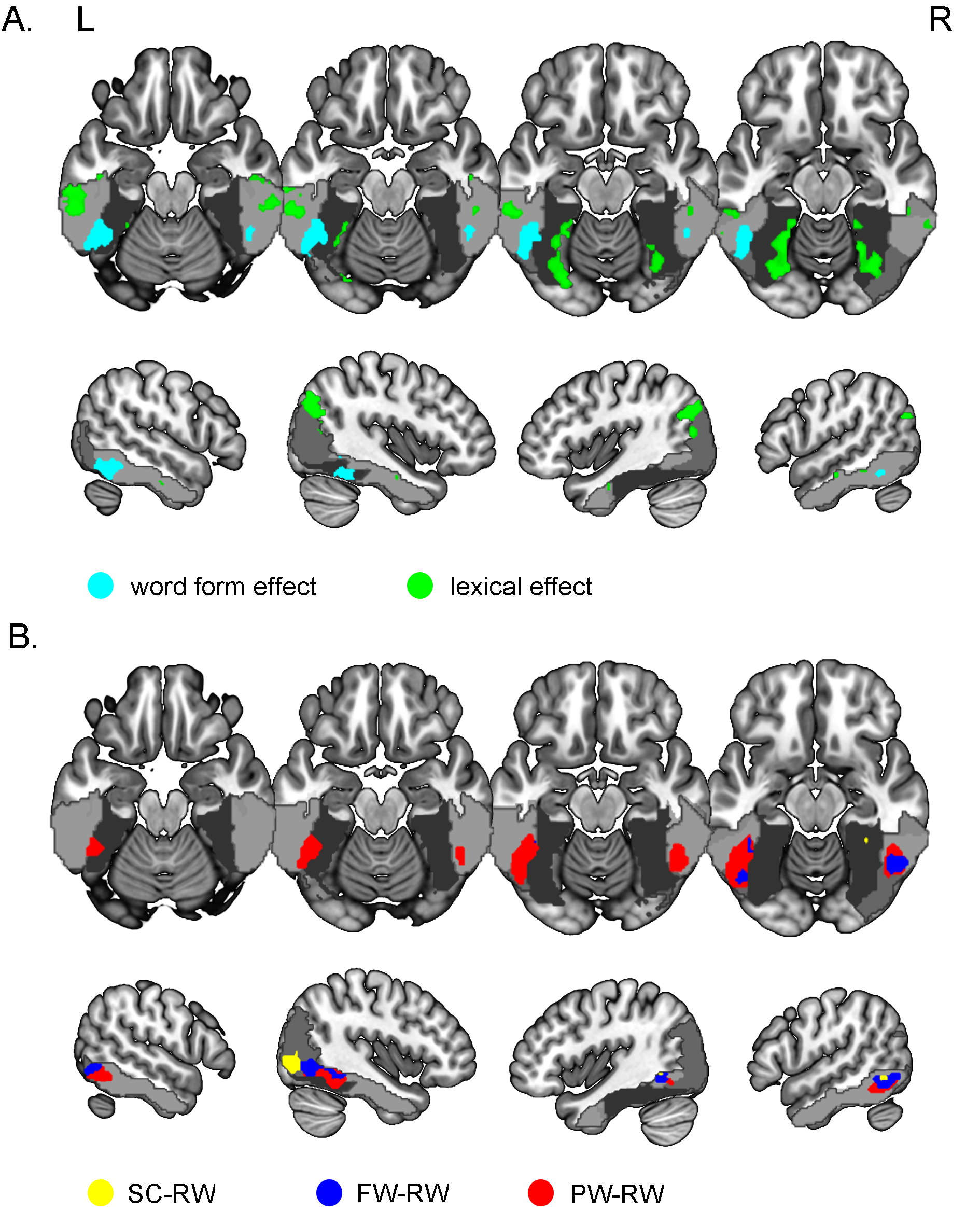

Supplement: Extended Data Figure 2-1 — Validation for functional activation results, supporting Figure 2. Download Figure 2-1, TIF file. [file enu-eN-CFN-0495-21-s01.tif]

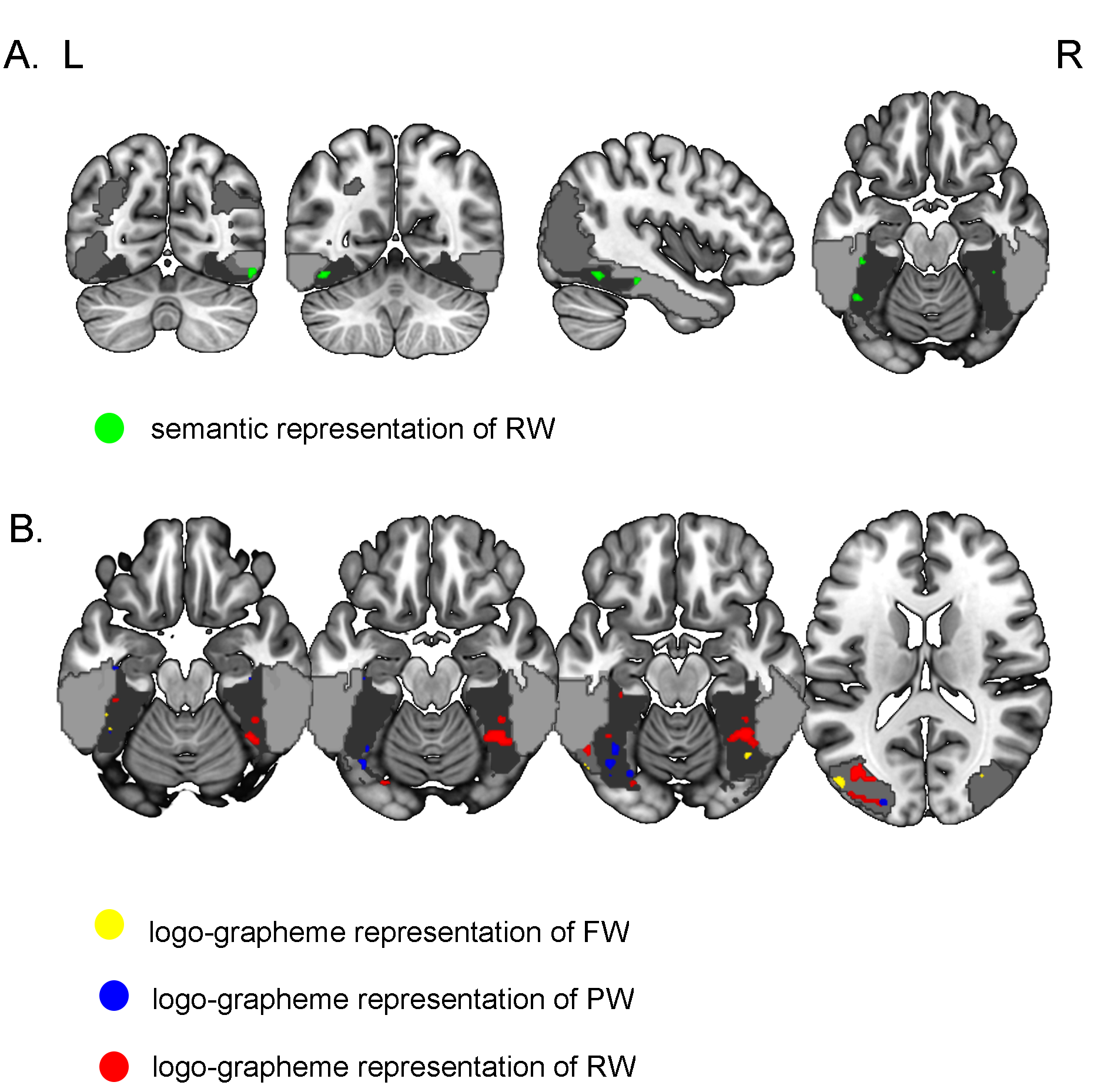

Supplement: Extended Data Figure 3-1 — Validation for RSA results, supporting Figure 3. Download Figure 3-1, TIF file. [file enu-eN-CFN-0495-21-s02.tif]

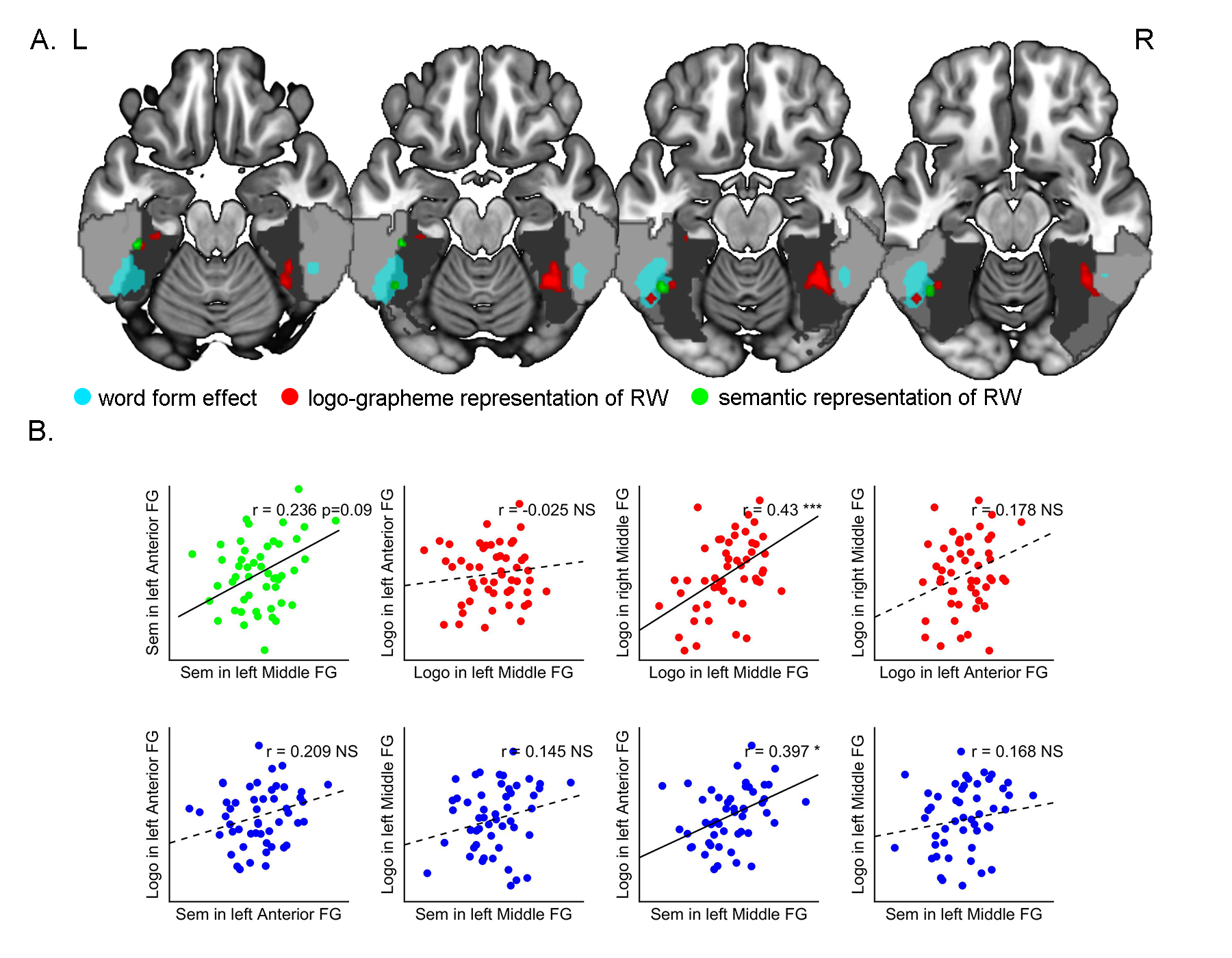

Supplement: Extended Data Figure 4-1 — Validation for RSA-behavior correlation results, supporting Figure 4. Download Figure 4-1, TIF file. [file enu-eN-CFN-0495-21-s03.tif]
