# Supplementary material for: Functional Gradient of the Fusiform Cortex for Chinese Character Recognition
Source: eNeuro. 2022 May 26;9(3):ENEURO.0495-21.2022. doi: 10.1523/ENEURO.0495-21.2022 (PMC9172282; doi:10.1523/ENEURO.0495-21.2022)
Supplement: Extended Data Table 1-1 — Main activation clusters and peaks of the lexical effect and word form effect as identified by contrasting RWs versus fixation minus PWs versus fixation and PWs versus fixation minus FWs versus fixation by removing trials with error response. This table is supporting the Table 1. Download Table 1-1, DOCX file. [file enu-eN-CFN-0495-21-s05.docx]

**Extended Table 1-1.** Main Activation Clusters and Peaks of the Lexical Effect and Word Form Effect as Identified by Contrasting RWs vs fixation Minus PWs vs fixation and PWs vs fixation minus FWs vs fixation by removing trials with error response. This table is supporting the Table 1.

| Index | Region | Cluster Size | Peak T value | Peak Coordinates | | | | |
| --- | --- | --- | --- | --- | --- | --- | --- | --- |
|  |  |  |  | x | | y | | z |
| Lexical Effect: RWs vs fixation minus PWs vs fixation | | | | | | | | |
| 1 | Right middle occipital gyrus, right middle temporal gyrus, right angular gyrus, right superior temporal gyrus | 159 | 5.93 | 44 | -74 | | 34 | |
| 2 | Left inferior temporal gyrus, left middle temporal gyrus, left fusiform gyrus | 158 | 5.80 | -58 | -26 | | -24 | |
| 3 | Left middle occipital gyrus, right middle temporal gyrus | 258 | 5.48 | -40 | -76 | | 38 | |
| 4 | Right inferior temporal gyrus, right fusiform gyrus, right middle temporal gyrus | 83 | 4.98 | 60 | -22 | | -26 | |
| 5 | Left middle temporal gyrus, left inferior temporal gyrus | 20 | 4.71 | -46 | -12 | | -24 | |
| 6 | Left fusiform gyrus, left inferior occipital gyrus, left middle occipital gyrus | 139 | 4.71 | -24 | -52 | | -16 | |
| 7 | Right fusiform gyrus | 33 | 4.70 | 22 | -64 | | -12 | |
| 8 | Left middle occipital gyrus | 49 | 4.70 | -18 | -90 | | 18 | |
| 9 | Right fusiform gyrus | 51 | 4.51 | 18 | -44 | | -12 | |
| 10 | Left middle temporal gyrus, left inferior temporal gyrus | 18 | 4.27 | -64 | -56 | | -6 | |
| 11 | Right middle occipital gyrus, right middle temporal gyrus | 14 | 3.90 | 40 | -72 | | 14 | |
| 12 | Right fusiform gyrus, right inferior occipital gyrus | 32 | 3.77 | 34 | -68 | | -14 | |
| 13 | Right superior occipital gyrus, right middle occipital gyrus | 10 | 3.54 | 34 | -80 | | 42 | |
| Word Form Effect: PWs vs fixation minus FWs vs fixation | | | | | | | | |
| 1 | Left middle occipital gyrus, left angular gyrus, left middle temporal gyrus, left superior occipital gyrus, left inferior parietal lobule | 365 | 6.34 | -40 | -80 | | 38 | |
| 2 | Right middle occipital gyrus, right middle temporal gyrus, right angular gyrus, right superior occipital gyrus, right inferior parietal lobule | 232 | 5.72 | 42 | -74 | | 34 | |
| 3 | Left inferior temporal gyrus | 12 | 4.05 | -58 | -28 | | -22 | |

**Extended Table 2-1.** Mean activations Clusters and Peaks as identified by Contrasting PWs vs fixation minus RWs vs fixation, FWs vs fixation minus RWs vs fixation, and SCs vs fixation minus RWs vs fixation by removing trials with error response. This table is supporting the Table 2.

| Index | Region | Cluster Size | Peak T value | Peak Coordinates | | | | |
| --- | --- | --- | --- | --- | --- | --- | --- | --- |
|  |  |  |  | x | | y | | z |
| Word-form orthographic processing: PWs vs fixation minus RWs vs fixation | | | | | | | | |
| 1 | Left inferior temporal gyrus, left inferior occipital gyrus, left fusiform gyrus**,** left middle temporal gurus, left middle occipital gyrus | 692 | 8.77 | -48 | -64 | | -10 | |
| 2 | Right inferior temporal gyrus, right fusiform gyrus, right middle temporal gyrus, right inferior occipital gyrus | 358 | 6.32 | 48 | -56 | | -10 | |
| 3 | Left middle occipital gyrus, left superior parietal lobule | 69 | 4.73 | -24 | -60 | | 42 | |
| 4 | Right middle occipital gyrus, right superior occipital gyrus | 19 | 4.56 | 30 | -66 | | 36 | |
| Radical orthographic processing: FWs vs fixation minus RWs vs fixation | | | | | | | | |
| 1 | Left inferior occipital gyrus, left middle occipital gyrus, left inferior temporal gyrus, left middle temporal gyrus, left fusiform gyrus | 407 | 6.03 | -46 | -68 | | -8 | |
| 2 | Right Inferior temporal gyrus, right middle occipital gyrus, right middle temporal gyrus, right inferior occipital gyrus | 303 | 5.79 | 46 | -56 | | -6 | |
| Basic visual processing: SCs vs fixation minus RWs vs fixation | | | | | | | | |
| 1 | Left middle occipital gyrus, left inferior occipital gyrus | 62 | 5.19 | -42 | -82 | | -4 | |

**Extended Table 3-1.** Clusters and Peaks for logo-grapheme and semantic representations of RWs, PWs and FWs in the vOT by removing trials with error response. This table is supporting the Table 3.

| Index | Region | Cluster Size | Peak Coordinates | | | | |
| --- | --- | --- | --- | --- | --- | --- | --- |
|  |  |  | x | | y | | z |
| Semantic Representations of RWs | | | | | | | |
| 1 | Left Fusiform Gyrus | 14 | -24 | -78 | | -4 | |
| 2 | Left Fusiform Gyrus | 27 | -44 | -56 | | -16 | |
| 3 | Right Fusiform Gyrus | 12 | 36 | -8 | | -36 | |
| 4 | Right Occipital Gyrus | 12 | 52 | -68 | | -14 | |
| Logo-grapheme Representations of RWs | | |  | | | | |
| 1 | Right Fusiform Gyrus | 67 | 36 | -54 | | -18 | |
| 2 | Left Middle Occipital Gyrus | 179 | -16 | -102 | | 2 | |
| 3 | Left Fusiform Gyrus | 38 | -32 | -60 | | -6 | |
| 4 | Left Middle Occipital Gyrus | 219 | -38 | -74 | | 14 | |
| 5 | Right Middle Occipital Gyrus | 43 | 36 | -72 | | 30 | |
| 6 | Left Fusiform Gyrus | 11 | -30 | -26 | | -24 | |
| 7 | Right Fusiform Gyrus | 11 | 38 | -44 | | -18 | |
| 8 | Left Inferior Occipital Gyrus | 13 | -50 | -62 | | -16 | |
| 9 | Left Middle Occipital Gyrus | 14 | -16 | -90 | | -6 | |
| 10 | Right Fusiform Gyrus | 11 | 34 | -48 | | -6 | |
| 11 | Right Middle Occipital Gyrus | 18 | 32 | -68 | | 24 | |
| Logo-grapheme Representations of PWs | | | | | | | |
| 1 | Left Middle Occipital Gyrus | 172 | -32 | -90 | | 26 | |
| 2 | Right Middle Occipital Gyrus | 100 | 36 | -94 | | -2 | |
| 3 | Left Middle Occipital Gyrus | 73 | -28 | -92 | | -4 | |
| 4 | Right Fusiform Gyrus | 19 | 32 | -41 | | -9 | |
| 5 | Right Fusiform Gyrus | 13 | 31 | -7 | | -32 | |
| 6 | Left Fusiform Gyrus | 11 | -38 | -70 | | -18 | |
| 7 | Left Fusiform Gyrus | 11 | -36 | -60 | | -16 | |
| 8 | Left Fusiform Gyrus | 12 | -26 | -74 | | -14 | |
| 9 | Right Middle Occipital Gyrus | 31 | 43 | -70 | | 26 | |
| 10 | Left Middle Occipital Gyrus | 10 | -28 | -86 | | 38 | |
| Logo-grapheme Representations of FWs | | | | | | | |
| 1 | Right Middle Occipital Gyrus | 84 | 42 | -80 | | 10 | |
| 2 | Right Fusiform Gyrus | 12 | 42 | -22 | | -28 | |
| 3 | Left Middle Occipital Gyrus | 10 | -36 | -82 | | 8 | |
| 4 | Left Middle Occipital Gyrus | 17 | -48 | -78 | | 14 | |
| 5 | Right Middle Occipital Gyrus | 12 | 30 | -80 | | 22 | |
| 6 | Right Superior Occipital Gyrus | 13 | 36 | -82 | | 42 | |
